# Neurointerface with oscillator motifs for inhibitory effect over antagonist muscles

**DOI:** 10.3389/fnins.2023.1113867

**Published:** 2023-03-24

**Authors:** Yulia Mikhailova, Anna Pozdeeva, Alina Suleimanova, Alexey Leukhin, Alexander Toschev, Timur Lukmanov, Elsa Fatyhova, Evgeni Magid, Igor Lavrov, Max Talanov

**Affiliations:** ^1^B-Rain Labs LLC, Kazan, Russia; ^2^Neuromorphic Computing and Neurosimulations Laboratory, Intelligent Robotics Department, Institute of Information Technologies and Intelligent Systems, Kazan Federal University, Kazan, Russia; ^3^Kazan Federal University, Kazan, Russia; ^4^Children's Republican Clinical Hospital, Ministry of Health of the Republic of Tatarstan, Kazan, Russia; ^5^School of Electronic Engineering, Tikhonov Moscow Institute of Electronics and Mathematics, HSE University, Moscow, Russia; ^6^Intelligent Robotics Department, Institute of Information Technologies and Intelligent Systems, Kazan Federal University, Kazan, Russia; ^7^Department of Neurology, Mayo Clinic, Rochester, NY, United States; ^8^Skolkovo Institute of Science and Technology, Moscow, Russia; ^9^Institute of Fundamental Medicine and Biology, Kazan Federal University, Kazan, Russia; ^10^Institute for Artificial Intelligence R&D, Novi Sad, Serbia

**Keywords:** neurointerface, neuromodulation, neurosimulation, spastic syndrome, oscillator motif, compensation, neurostimulation, neuroprosthesis

## Abstract

The effect of inhibitory management is usually underestimated in artificial control systems, using biological analogy. According to our hypothesis, the muscle hypertonus could be effectively compensated *via* stimulation by bio-plausible patterns. We proposed an approach for the compensatory stimulation device as implementation of previously presented architecture of the neurointerface, where (1) the neuroport is implemented as a DAC and stimulator, (2) neuroterminal is used for neurosimulation of a set of oscillator motifs on one-board computer. In the set of experiments with five volunteers, we measured the efficacy of motor neuron inhibition *via* the antagonist muscle or nerve stimulation registering muscle force with and without antagonist stimulation. For the agonist activation, we used both voluntary activity and electrical stimulation. In the case of stimulation of both the agonist and the antagonist muscles and nerves, we experimented with delays between muscle stimulation in the range of 0–20 ms. We registered the subjective discomfort rate. We did not identify any significant difference between the antagonist muscle and nerve stimulation in both voluntary activity and electrical stimulation of cases showing agonist activity. We determined the most effective delay between the stimulation of the agonist and the antagonist muscles and nerves as 10–20 ms.

## 1. Introduction

The range of diseases that cause the development of a spastic syndrome is quite large, and the most common causes are stroke, traumatic brain injury and spinal cord injury and demyelinating diseases, central nervous system (CNS) tumors, and some inflammatory and neurodegenerative diseases that impact the upper motor neurons (Rizzo et al., 2004; Zorowitz et al., 2013). There are more than 12 million people suffering from spastic syndrome as a consequence of some neurological conditions with a disability of approximately 12–27% (Kilgore, 2015). The spastic syndrome affects 20–40% of the stroke survivors, 65–78% of the patients with spinal cord injury, and 85% of the patients with multiple sclerosis. As a result, spastic syndrome leads to a disability in 7 years after the onset of the disease in 25% of the cases. More than half of the patients with stroke have various severities of movement disorders that are often manifested in an increased muscle tone as spasticity that significantly reduces the quality of life and often leads to a permanent disability (Livshits et al., 2002; Rizzo et al., 2004; Zorowitz et al., 2013). The increased frequency of congenital and perinatal pathology of the CNS in the population, owing to the development of medical technology, causes the prevalence of cerebral palsy (CP). The nursing of extremely premature babies causes their growth as patients with a severe perinatal damage to brain structures. Cerebral palsy is accompanied by motor disorders that occur in 100% of the cases, speech disorders in 75%, mental disorders in 50%, and sensory disorders in 25% (Evseev et al., 2010).

Spastic syndrome is manifested by a periodic or a regular involuntary hyperactivity of the skeletal muscles, the cause of which is attributed to a violation of the signal from the central nervous system to the muscles. The functional rewiring of the nervous system, leading to some compensation of the spastic syndrome, is a process that spreads widely from the spinal level to the supraspinal structures. Clinical symptoms have several phases such as: (1) muscle weakness formation, (2) spastic syndrome formation, and (3) recovery (Kovalenko et al., 2021). The appearing spastic syndrome is a sequential process that goes through all the aforementioned phases at different speeds. The muscle weakness formation phase gets developed during the acute period of a brain injury and is usually accompanied with deep reflexes decay, pareses development, and muscle hypertonus formation. The muscle force is regulated by corticospinal tract decays during impairment of the upper motor neurons of any etiology due to a supraspinal control impairment of the muscle tonus. The suprasegmental influences impairment and denervation of the α-motor neurons that triggers the restructuring of the segmental apparatus (Trompetto et al., 2014). The spastic syndrome formation phase is accompanied with a reorganization of the brain circuits and usually gets manifested during the 1–6 weeks after a CNS injury. This phase includes the development of hyperexcitability of the α-motor neurons due to impairment of the afferent signal processing that facilitates Ia signaling at segmental level (Delwaide and Oliver, 1988; Nakashima et al., 1989; Wilson et al., 1999). During this phase, a gradual increase in spastic syndrome, deep reflex and clonuses, extensor and flexor contractions, and synkineses commonly happens. Further changes during the final recovery phase manifest with a decrease in spastic syndrome, synergical voluntary motions, restoration of the complex movements, and restoration of the normal function with voluntary locomotion (Levin et al., 2009; Bestmann et al., 2010; Madhavan et al., 2011). A current understanding of the key phases represents spastic syndrome as a delayed compensation to a complex plastic, structural, and functional reorganization of the CNS and the muscles. The motoneurons of the antagonist muscles are reciprocally inhibited *via* the Ia interneurons that are activated by an Ia afferent of the muscle antagonist (Guertin, 2013; Côté et al., 2018). The treatment of patients with spastic syndrome depends on several details such as: the nature of the cause of the disease and disease progression, adequacy of the treatment, accessibility of the treatment, and rehabilitation potential of the patient. The rehabilitation potential of the patient is closely related to concomitant conditions, such as the presence and severity of pain syndrome, bedsores and infectious processes, and orthopedic pathology of the extremities.

The existing methods of treatment for spastic syndrome yield varying results and require a further subject-specific study. The spectrum of these methods ranges from the intake of antipathetic oral medication to destructive interventions in the spinal cord (Iskra et al., 2018). However, improvement in motor functions is not observed in all patients, and often in ambulatory patients, it even leads to a temporary decrease in standing and walking activity. When conservative methods of rehabilitation have no effect neurosurgical operations are often used (Lazorthes et al., 2002). Surgical methods are also varied and do not have strict algorithms. Such surgical methods include, for example, the use of intrathecal baclofen delivery with programmable pumps (Albright, 1992), neuromodulation, selective dorsolateral rhizotomy (El-Hefnawy et al., 2015), and orthopedic surgery (Aboutorabi et al., 2017). In case of lack of any noticeable effect, various destructive interventions are recommended. Stereotactic interventions on the brain and posterior longitudinal myelotomy are performed during the treatment of severe forms of spasticity. These interventions in the form of operations are rarely performed due to possible complications, therefore, they have not been employed widely in clinical practice, and thus, we assume that there is no standardized approach for the treatment of spasticity.

In this article, we use our earlier presented approach of the neurointerface (Talanov et al., 2021) to compensate for the spastic syndrome. We used the bio-plausible pattern generated with the set of oscillator motifs (OMs) to trigger the antagonist (to spastic muscle) nerve that stimulates the inhibitory projection of the agonist (spastical) muscle Ia nuclei to test the hypothesis of an effective inhibition of neuronal activity, thus muscle activity. We measured the agonist muscle force in both cases of the antagonist muscle and the nerve stimulation.

## 2. Subjects and methods

### 2.1. The study setup

**Figure 1:**
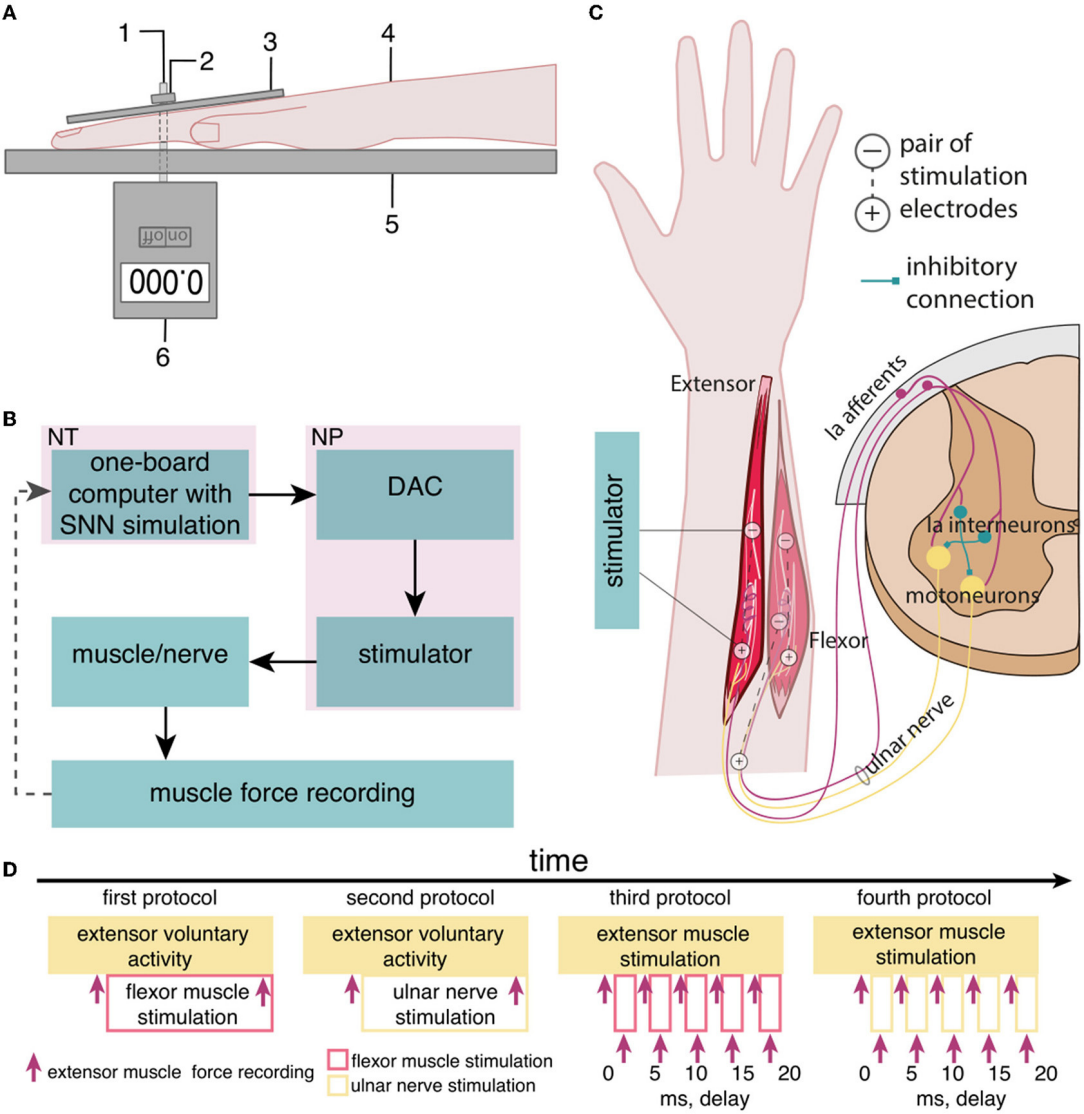
The setup of the neurointerface for muscle stimulation and muscle force recording. **(A)** (1) Fasteners, (2) the clamp, (3) the upper plate, (4) the volunteer's hand, (5) the lower plate, and (6) the dynamometer; **(B)** the setup of the neurointerface where NT is the neuroterminal and NP is the neuroport; and **(C)** the diagram of stimulation: (1) activated muscles and nerve, (2) placement of electrodes [the antagonist muscle (flexor) inhibits the extensor *via* reflex arc]; and **(D)** the timeline of the study.

**Figure 2:**
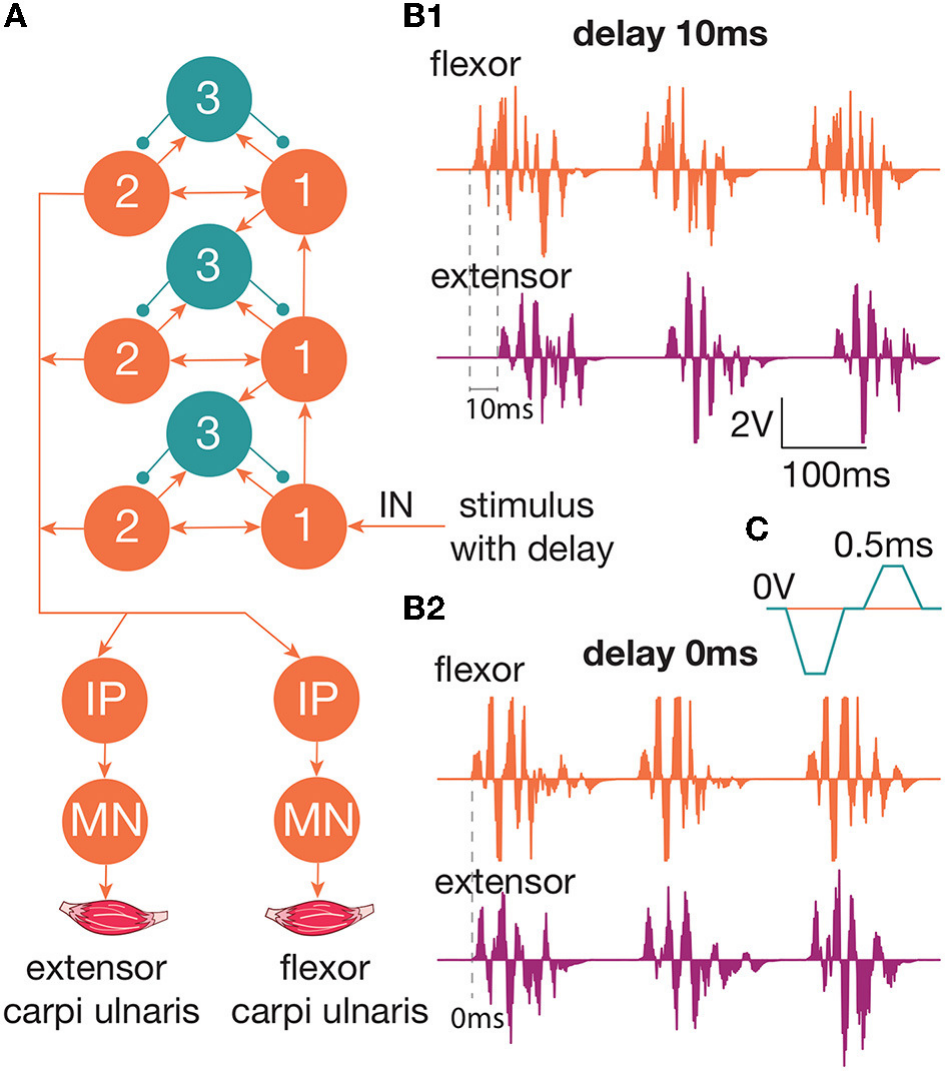
**(A)** The diagram of the circuit that produces neuronal activity to trigger the muscle/nerve. This circuit is used for the generation of activity in a spiking neural network. We used a three-layered OM network with a 20-Hz stimulation of the first group of neurons in the first layer. Outputs arrive on the motoneurons of the muscles—extensor carpi ulnaris and flexor carpi ulnaris. **(B1)** The produced spiking activity with a delay of extensor activity generation 10 ms relative to the flexor. The extensor muscle activity starts after the flexor activity. There are two impulses per 100 ms, which indicate 20-Hz impulses **(B2)**. The produced spiking activity with a delay of extensor activity generation 0 ms relative to flexor. Both the muscles are active at the same time. **(C)** The modulation of 2 kHz. Every impulse should be reset to zero voltage every 0.5 ms.

We studied the inhibitory effect of the extensor muscle activity on the muscles: the ulnar extensor carpi (*Musculus extensor carpi ulnaris*) and the antagonist ulnar flexor carpi (*Musculus flexor carpi ulnaris*). Then, we studied the inhibition of the extensor muscle with activation of the ulnar nerve, which innervates the flexor and also includes the Ia afferent of the flexor. We assume that activation of the flexor or the ulnar nerve triggered the inhibitory circuit with Ia interneurons (Figure 1C). There are several articles dedicated to the spastic syndrome compensation using the noninvasive electrical nerve stimulation (Perez et al., 2003; Karakoyun et al., 2015; Lu et al., 2020). More than that, attempts to compensate for spastic syndrome have led to the development of several devices (Palmcrantz et al., 2020; Pennati et al., 2021). In this study, we used the previously proposed approach for the neurointerface (Talanov et al., 2021) using oscillator motifs to generate bio-plausible neural activity (Talanov et al., 2020). We used a neuron circuit that consists of OMs to produce a bio-plausible pattern of neural activity. A schematic description of the study setup is presented in Figure 1B. The hardware devices to stimulate the muscle or the nerve are neuroports where the software that implements a spiking neural network is a neuroterminal (Talanov et al., 2021). The one-board computer generates the neuronal activity in real-time neurosimulation. The OM (Figure 2A) is a basic unit of the model and produces various durations of activity (Talanov et al., 2020) that depends on the balance of excitatory and inhibitory weights. We used the neuronal circuit with 3 OMs (Figure 2B) for stimulation since previously we had indicated that the 3 OM circuit is the most comfortable neuronal circuit for the participating volunteer (Talanov et al., 2021). The discomfort rate was estimated by the subjective feeling of the volunteers. The discomfort rate of 1 denotes no pain and 10 denotes the maximum tolerable level of pain. This circuit was activated by a stimulus with 20-Hz frequency. The generated signal was modulated with 2 kHz. This modulation is shown in Figure 2C. The generated activity was transmitted *via* DAC to the stimulator that triggers the muscle or the nerve (Figure 1B). To assess the effect of inhibition, we recorded the muscle force during voluntary activity and stimulation as well as during the inhibitory effect produced by the antagonist's muscle or nerve activation (Figure 1A). The study setup for recording the muscle force included: the lower plate, the upper plate, fasteners, the clamp, and the dynamometer (Figure 1A). The volunteer's hand, palm down, was placed on the lower plate (15 x 30 cm) with the hole in the left side. The top plate (4 x 14 cm) with the hole in the left side was placed above the palm. Two plates were connected with the fastener that was located between the middle and ring fingers of the volunteer. The clamp of the fastener located on the upper plate was used to change the distance between the plates to securely fix a volunteer's hand. Thus, during the increase in muscle force, the immobilized palm pressed on the top plate that pulled the dynamometer lever indicating the change of force.

### 2.2. Research involving humans and animals rights statement

Five healthy volunteers participated (3 male, 2 female, age: 23 ± 2) in the current study.

### 2.3. Informed consent

All participants gave an informed written consent to participate in the study, in accordance with the Declaration of Helsinki, and were introduced to the study protocol.

### 2.4. The stimulation protocols

For non-invasive stimulation, we used two hypoallergenic reusable gel electrodes VUPIESSE 32 mm × 32 mm that we placed in the projection of the flexor muscle at a distance of 5–8 cm from each other (Figure 1C).

We used four protocols of the study (Figure 1D): (1) the voluntary extension with the ulnar flexor muscle stimulation, (2) the voluntary extension with the ulnar nerve stimulation because this nerve includes efferents and afferents of the antagonist muscle (flexor), (3) the stimulation of the extensor and flexor muscles with delays, and (4) the stimulation of the extensor muscle and the ulnar nerve with delays. During the first phase of the study, we had recorded the muscle force (Figures 1A, D) of the participant's voluntary activity when the volunteer unbended the palm at a comfortable level of muscle tension and maintained this position throughout the study protocol. During the second phase, we turned on the flexor muscle stimulation, adjusting the stimulation current according to the volunteer's comfort level and subjective assessment of the decrease in muscle extension. We recorded the muscle force of the extensor with flexor stimulation.

For the second protocol, we stimulated the ulnar nerve that triggers Ia afferents of the antagonist instead of the flexor muscle in particular: stimulation electrodes were placed on the ulnar nerve: the cathode was placed at the cubital canal and the anode at the projection of the ulnar flexor muscle.

We used the third protocol (Figure 1D) to identify and measure the most effective delays between the stimulation of the flexor and extensor muscles (0, 5, 10, 15, and 20 ms). We assume that if the flexor (antagonist) is stimulated earlier than the extensor (agonist), the activity of the agonist is reduced through the inhibitory interneuronal pool in the spinal cord. We located the electrodes for transcutaneous stimulation of the flexor and the extensor in the projection of the muscles at a distance of 5–8 cm between cathode and anode. We stimulated the agonist muscle by increasing the current to a comfortable level for the volunteer and recorded the muscle force. Later, we stimulated the antagonist muscle with an earlier signal relative to the agonist with a preset delay and recorded the muscle force. In the case of the fourth protocol, we ran the stimulation similarly to the third protocol, except for the antagonist ulnar nerve instead of the flexor muscle.

## 3. Results

**Figure 3:**
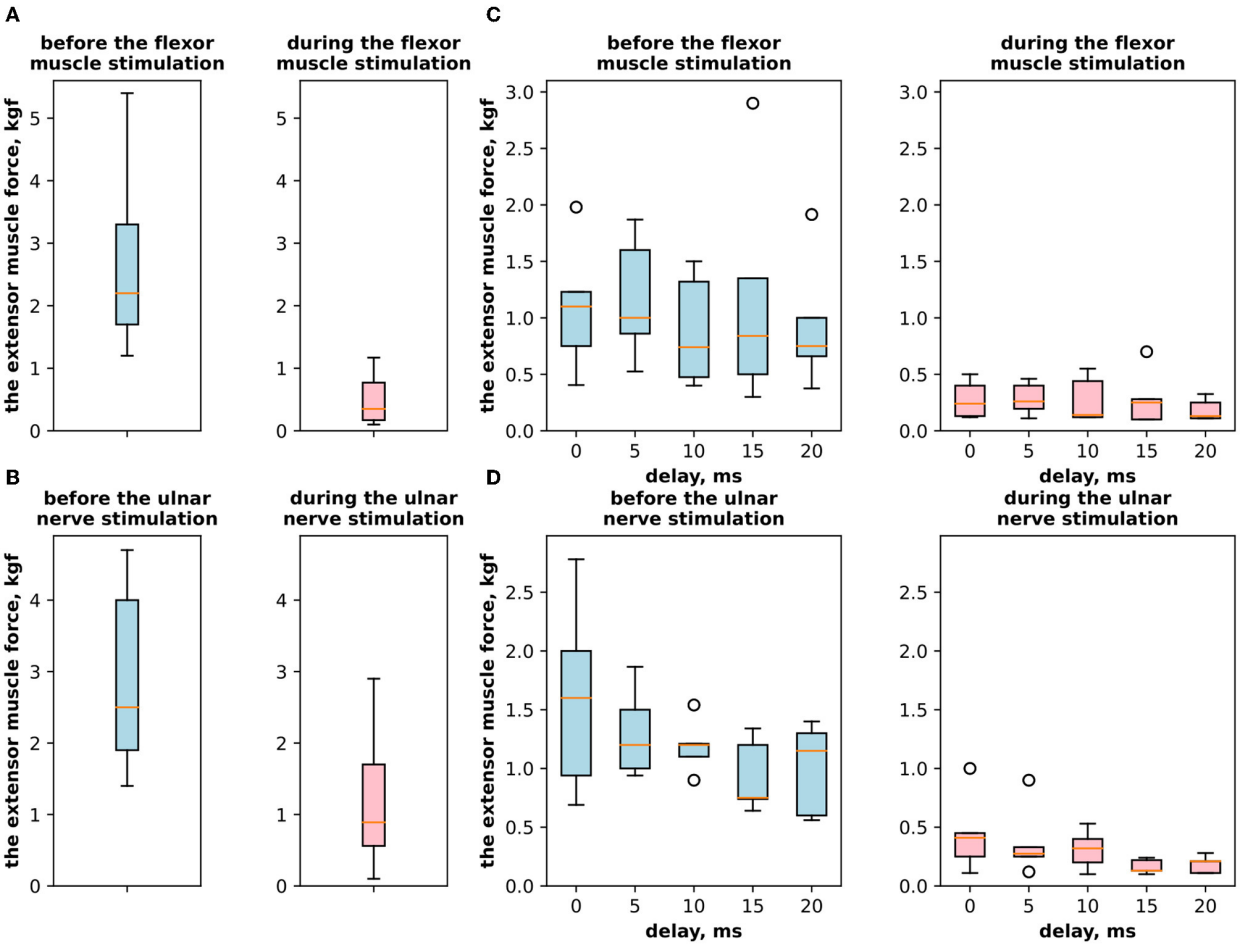
**(A)** The extensor muscle force with voluntary activity and during antagonist muscle stimulation (inhibitory effect). **(B)** The extensor muscle force with voluntary activity and during the ulnar nerve stimulation. **(C)** The extensor muscle force before antagonist muscle stimulation and during antagonist muscle stimulation (inhibitory effect) with the range of delays. **(D)** The extensor muscle force before antagonist nerve stimulation and during the ulnar nerve stimulation that innervates the flexor and triggers Ia afferent of the flexor, with the range of delays.

**Figure 4:**
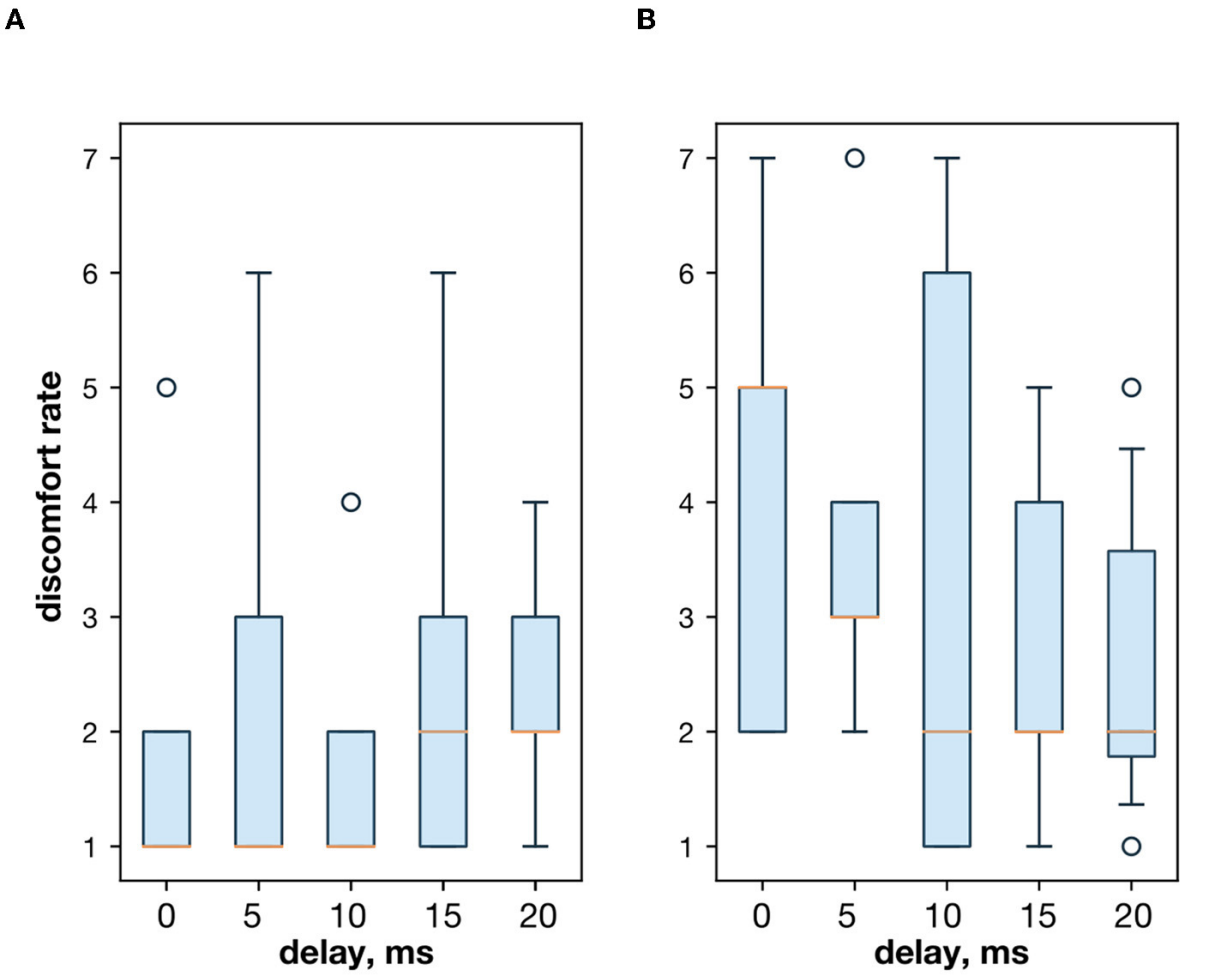
**(A)** The discomfort rate with the extensor and flexor muscle stimulation. **(B)** The discomfort rate with the extensor muscle and the ulnar nerve stimulation.

**Figure 5:**
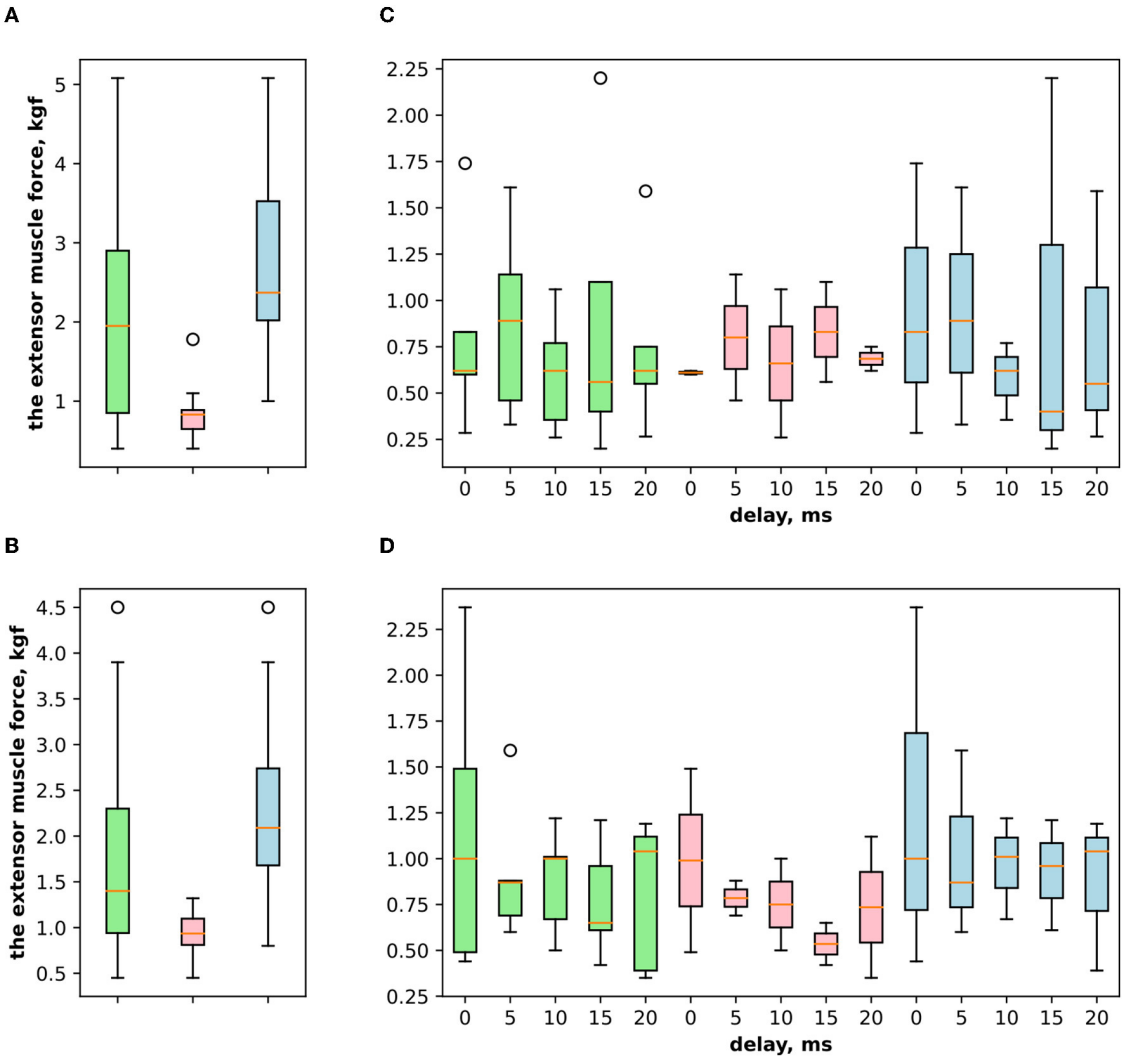
**(A)** The extensor muscle force difference between voluntary activity and during antagonist muscle stimulation (inhibitory effect), where the green boxplot reflects the delta for both genders of volunteers (women and men) and pink reflects the delta for women and blue for men. **(B)** The extensor muscle force difference between voluntary activity and during the ulnar nerve stimulation (inhibitory effect). **(C)** The extensor muscle force difference between before antagonist muscle stimulation and during antagonist muscle stimulation (inhibitory effect) with the range of delays. **(D)** The extensor muscle force difference before antagonist nerve stimulation and during the ulnar nerve stimulation that innervates the flexor and triggers Ia afferent of the flexor, with the range of delays.

First, we studied the inhibitory effect produced by the antagonist muscle (flexor) or the ulnar nerve stimulation during the voluntary extension. We recorded the extensor muscle force during voluntary activity. The average muscle force was 2.5 ± 1.1 kgf (Figure 3A). Then, we stimulated the antagonist muscle and the extensor muscle force decreased significantly to 0.48 ± 0.33 kgf (*p* < 0.05) (Figure 3A). The registered subjective discomfort rate was 2.3 ± 1.1 out of 10, where 10 denotes the maximum discomfort. Furthermore, we conducted studies with the ulnar nerve stimulation during voluntary extension. The average muscle force during voluntary activity was 2.85 ± 0.13 (Figure 3B). Then, we stimulated the ulnar nerve that includes afferents and efferent fibers of the flexor muscle and the extensor muscle force decreased considerably to 1.1 ± 0.8 kgf (*p* < 0.05) (Figure 3B). The discomfort rate was 2.9 ± 1.6 out of 10. The antagonist muscle stimulation had a stronger inhibitory effect on the extensor muscle force decrease than the ulnar nerve stimulation (0.48 ± 0.33 kgf vs. 1.1 ± 0.8 kgf, *p* < 0.05). The voltage of the flexor muscle stimulation was higher than that of the ulnar nerve stimulation (15.2 ± 4.8 V vs. 11.2 ± 2.9 V, *p* < 0.1) and because the nerve stimulation influenced the muscle directly, less voltage was required for activation. In the second series of studies, we researched the inhibitory effect produced by the activation of the antagonists' muscles with the range of delays (0–20 ms with a 5 ms step) between extensor and flexor stimulation. The results of the extensor inhibition with flexor muscle stimulation are presented in Table 1. The extensor muscle force decreased significantly with flexor muscle stimulation (*p* < 0.05). The strongest drop in the extensor muscle force to 0.19 ± 0.09 kgf was recorded in the study with a 20 ms delay (Figure 3C). The discomfort rate in this experiment was 2.4 ± 1.1 out of 10 (Figure 4A). The most comfortable mode (1.8 ± 1.3) for the antagonist's muscle stimulation was observed with a 10 ms delay. This mode also demonstrated a significant decrease in the extensor muscle force from 0.89 ± 0.5 to 0.27 ± 0.2 (Figure 3C). The extensor muscle force declined in the mode with stimulation of the antagonists' muscles without delay from 1.09 ± 0.93 kgf to 0.28 ± 0.17 kgf (Figure 3C). Then, we researched the inhibitory effect produced by the ulnar nerve stimulation. The ulnar nerve innervates the antagonist muscle (flexor) and Ia afferents of the antagonist, inhibiting the motor neuron of the agonist muscle (extensor). Similar to a previous study with the antagonists' muscle activation, we stimulated the extensor muscle and the ulnar nerve with different delays (0–20 ms). The results (Table 2) demonstrated a significant decrease in the extensor muscle force with the ulnar nerve stimulation (*p* < 0.05) regardless of delays. The most significant difference (*p* < 0.01) was observed with a 10 ms delay between the ulnar nerve stimulation and the extensor muscle stimulation. In this mode, the extensor muscle force decreased from 1.19 ± 0.23 kgf to 0.31 ± 0.17 kgf (Figure 3D). The discomfort rate was 3.4 ± 2.9 out of 10 (Figure 4B). The most comfortable mode (2.4 ± 1.5) with the ulnar nerve stimulation was recorded with a 20 ms delay. In this mode, the extensor muscle force also decreased considerably from 1.0 ± 0.4 to 0.18 ± 0.07 (Figure 3D). The highest discomfort rate was (4.2 ± 2.2) in the mode without delay between the ulnar nerve stimulation and the extensor muscle stimulation. The extensor muscle force decreased in the mode without delay from 1.6 ± 0.84 kgf to 0.44 ± 0.34 kgf (Figure 3D). The voltage for the extensor activation was consistent across all studies at 25 ± 4 V (Tables 1, 2), whereas for the flexor muscle stimulation, the voltage was slightly higher (11.8 ± 3 V) than that for the ulnar nerve stimulation (9.4 ± 3.5 V; *p* < 0.1). The average discomfort rate was insignificantly lower with the flexor muscle stimulation (2.2 ± 1.6) than with the ulnar nerve stimulation (3.3 ± 2.0). There was no significant difference (*p* > 0.1) in the extensor muscle force with the flexor muscle stimulation or the ulnar nerve stimulation. We failed to find any significant difference between male and female volunteers' responses triggered by muscle or nerve stimulation. These results are reflected in Figure 5. Figure 5A reflects the deltas between volunteer activity and the antagonist muscle stimulation. Figure 5B reflects the deltas between volunteer activity and the ulnar nerve stimulation. Figure 5C reflects the deltas between volunteer activity and the antagonist muscle stimulation splitted by delays. Figure 5D reflects the deltas between volunteer activity and the ulnar nerve stimulation splitted by delays. In all subfigures It is shown in force before and after the stimulation, green color is used to indicate deltas for both genders simultaneously, pink color reflects deltas in force for women, and blue for men.

**Table 1:** Experimental results for stimulation of the extensor and flexor muscles.

	**0 ms**	**5 ms**	**10 ms**	**15 ms**	**20 ms**
Flexor voltage, V	13.2 ± 4	12 ± 3	11 ± 2.6	11.4 ± 2.6	11.6 ± 3.2
Extensor voltage, V	25.4 ± 4.6	26.4 ± 3.5	25.4 ± 4.6	25 ± 4.1	25.8 ± 5.1
Extensor force, kgf, mean ± std	1.09 ± 0.93	1.17 ± 0.55	0.88 ± 0.5	1.17 ± 1	0.94 ± 0.59
Extensor force with inhibition, kgf, mean ± std	0.27 ± 0.17	0.28 ± 0.14	0.27 ± 0.20	0.28 ± 0.24	0.18 ± 0.09

**Table 2:** Experimental results for stimulation of the extensor muscle and the nerve.

	**0 ms**	**5 ms**	**10 ms**	**15 ms**	**20 ms**
Flexor voltage, V	10 ± 3.5	9.2 ± 3.7	9.2 ± 3.7	9.4 ± 4.4	9.4 ± 3.8
Extensor voltage, V	25.4 ± 4.6	25.4 ± 4.6	25.4 ± 4.6	25.4 ± 4.6	25.4 ± 4.6
Extensor force, kgf, mean±std	1.60 ± 0.84	1.30 ± 0.38	1.19 ± 0.23	0.93 ± 0.31	1.00 ± 0.40
Extensor force with inhibition, kgf, mean±std	0.44 ± 0.34	0.37 ± 0.30	0.31 ± 0.17	0.16 ± 0.06	0.18 ± 0.07

## 4. Conclusion

Both the antagonist muscle and nerve stimulation had a reduction effect and significantly decreased the extensor muscle force. The subjective perception of volunteers was different for the antagonist muscle and the ulnar nerve stimulation. If the two muscles' stimulation produced the counterforce effect, then the stimulation of the ulnar nerve produced the inhibitory effect, thereby decreasing the force in the agonist muscle while keeping the subjective activation from the brain. The discomfort rate was slightly higher with the nerve stimulation but insignificant. The subjective perception of volunteers that the nerve stimulation is more focused than the muscle stimulation, thus the discomfort rate is higher. The voltage for the flexor muscle stimulation was slightly higher than that for the ulnar nerve because the nerve stimulation more directly triggered the muscle. The antagonist muscle stimulation had a stronger muscle force reduction effect than the ulnar nerve stimulation during voluntary extension. That might be connected to all factors, such as higher voltage, lower discomfort rate, and effect on the muscle. However, in control when the extensor (agonist) muscle was activated with the same value during the study, we did not observe a significant difference in the reduction effect on the extensor muscle force with the flexor muscle stimulation or the ulnar nerve stimulation. We also noticed that with a delay between the flexor muscle/ulnar nerve and the extensor muscle stimulation, the muscle force decreased to lower values (Tables 1, 2). We assume that during the late stimulation of the extensor (10–20 ms), its motoneurons were already partially inhibited by the antagonist Ia afferents, influence. We have to admit that the simultaneous stimulation of agonist and antagonist muscles or nerves decreased the extensor (agonist) muscle force significantly (1.09 ± 0.93 kgf to 0.28 ± 0.17 kgf and 1.6 ± 0.84 kgf to 0.44 ± 0.34 kgf). In future, we plan to extend the research with more participants, compared with traditional in neurorehabilitation domain monophasic and biphasic square pulses stimulation for both agonist and antagonist muscles. The authors suppose that there is an interesting option to close the loop taking into account the EMG as the feedback loop to set up the electrical stimulation pattern formation and use the estimate of muscle reciprocity with the method described in Lobov et al. (2018).

## Data availability statement

The original contributions presented in the study are included in the article/supplementary material, further inquiries can be directed to the corresponding author.

## Ethics statement

The study was approved by the Kazan Federal University Ethics Committee (Reference 12). The patients/participants provided their written informed consent to participate in this study.

## Author contributions

YM, AP, AS, AL, EF, and TL experiments and results processing. IL and EF medical part of work management. AT and EM engineering part of work management. MT overall management of the work. EF, AS, and MT introduction. All authors contributed to the article and approved the submitted version.
